# The Utilization and Interpretation of Cardiac Biomarkers During Pregnancy: *JACC: Advances* Expert Panel

**DOI:** 10.1016/j.jacadv.2022.100064

**Published:** 2022-08-26

**Authors:** Amy A. Sarma, Niti R. Aggarwal, Joan E. Briller, Melinda Davis, Katherine E. Economy, Afshan B. Hameed, James L. Januzzi, Kathryn J. Lindley, Deirdre J. Mattina, Brandon McBay, Odayme Quesada, Nandita S. Scott, American College of Cardiology Cardiovascular Disease in Women Committee and Cardio-obstetrics Work Group

**Affiliations:** a Massachusetts General Hospital, Boston, Massachusetts, USA; b Harvard Medical School, Boston, Massachusetts, USA; c Department of Cardiovascular diseases, Mayo Clinic, Rochester, Minnesota, USA; d Division of Cardiology, University of Illinois at Chicago, Chicago, Illinois, USA; e Division of Cardiovascular Medicine, University of Michigan, Ann Arbor, Michigan, USA; f Brigham and Women’s Hospital, Boston, Massachusetts, USA; g University of California, Irvine, Orange, California, USA; h Baim Institute for Clinical Research, Boston, Massachusetts, USA; i Cardiovascular Division, Washington University in St. Louis, St. Louis, Missouri, USA; j Division of Cardiovascular Medicine, Cleveland Clinic, Cleveland, Ohio, USA; k Women’s Heart Center, The Christ Hospital Heart and Vascular Institute, Cincinnati, Ohio, USA; l The Carl and Edyth Lindner Center for Research and Education, The Christ Hospital, Cincinnati, Ohio, USA

**Keywords:** biomarker, heart failure, natriuretic peptide, pregnancy, troponin

## Abstract

Cardiac biomarkers are widely used in the nonpregnant population when acute cardiovascular (CV) pathology is suspected; however, the behavior of these biomarkers in the context of pregnancy is less well understood. Pregnant individuals often have symptoms that mimic those of cardiac dysfunction, and complications of pregnancy may include CV disease. This paper will summarize our current knowledge on the use of cardiac biomarkers in pregnancy and provide suggestions on how to use these tools in clinical practice based on the available evidence. Natriuretic peptides and troponin should not be measured routinely in uncomplicated pregnancy, where values should remain low as in the nonpregnant population. In the context of pre-existing or suspected CV disease, these biomarkers retain their negative predictive value. Elevations of both natriuretic peptides and troponin may occur without clear clinical significance in the immediate postpartum period. Elevations of these markers should always prompt further investigation into possible CV pathology.

## Highlights

• Cardiac biomarker testing is widely utilized in the nonpregnant population; however, their use in the context of pregnancy is less well understood.
• Cardiac biomarkers, particularly natriuretic peptides, retain their negative predictive value during pregnancy.
• Biomarker elevations should prompt investigation into cardiac pathology but can occur immediately postpartum without apparent consequence.
• Several novel biomarkers remain of interest in pregnancy and merit further investigations.

Pregnancy is associated with increased hemodynamic demands on the maternal cardiovascular system. Significant neurohormonal fluctuations contribute to alterations in the cardiovascular system, which allow adaptation to the increased demands of normal pregnancy. Pregnant individuals often have symptoms of fatigue, dyspnea, decreased exercise capacity, and lower extremity edema in the context of normal pregnancy that can be challenging to distinguish from those of cardiac dysfunction. Furthermore, complications of pregnancy may include cardiovascular manifestations such as cardiomyopathy. While cardiac biomarker testing is widely utilized for diagnosis and monitoring of patients with suspected or proven cardiovascular disease, their use in the context of pregnancy is less well understood. Therefore, a better understanding of whether and how cardiac biomarkers can be integrated into clinical practice provides an important means of identifying pregnant individuals who require further cardiovascular evaluation. This paper will summarize our current knowledge on the use of cardiac biomarkers in pregnancy and provide suggestions on how to optimally use these tools in clinical practice based on the available evidence.

## Hemodynamics changes of pregnancy

**Figure 1:**
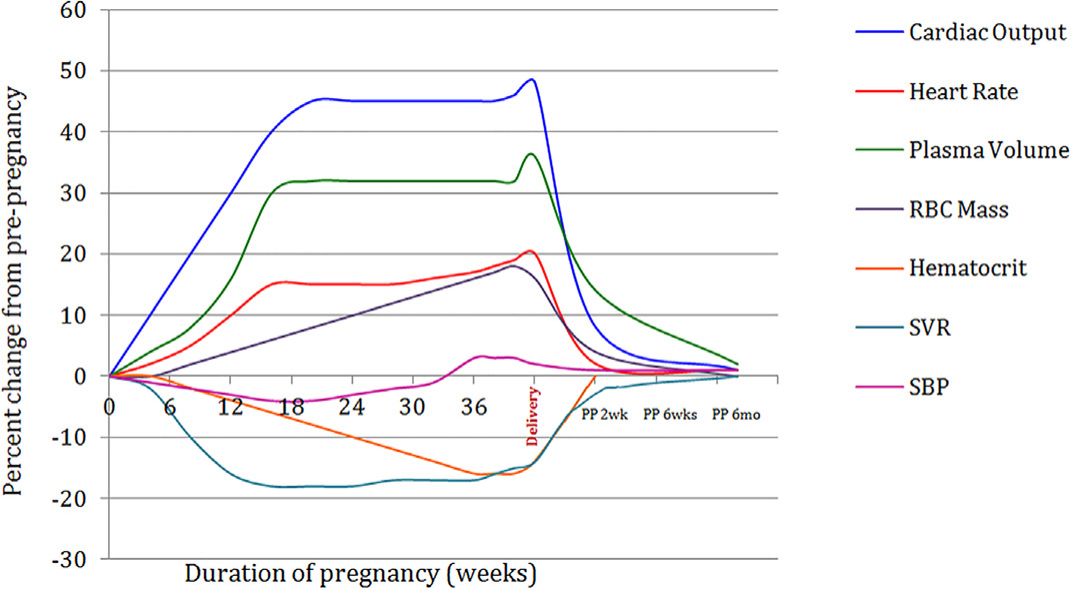
**Normal Cardiovascular Adaptations of Pregnancy**^2^ A rise in plasma volume and heart rate result in a 30% to 50% increase in cardiac output. Systemic vascular resistance (SVR) falls associated with a drop in systolic blood pressure (SBP) that rises toward normal nearer term. Red blood cell (RBC) mass increases, though to a lesser extent than plasma volume, resulting in a dilutional anemia with fall in hematocrit. These adaptations are amplified in individuals with multiple gestations. Reprinted by permission from Springer Nature: *Current Treatment Options in Cardiovascular Medicine* Pregnancy in Women with Congenital Heart Disease. 19(9):73 [copyright] 2017.

The normal cardiovascular hemodynamic adaptations to pregnancy include a rise in plasma volume, heart rate and thus cardiac output (CO), and a decline in peripheral vascular resistance as shown in Figure 1.^1^^,^^2^ During labor and delivery, CO is increased further due to increased venous return from the contracting uterus as well as an increase in the heart rate. Immediately postpartum, auto transfusion of blood from uterine involution, increased venous return due to decreased IVC compression, and rapid mobilization of dependent edema all result in increased CO. Afterload increases due to the loss of the low vascular resistance placental unit. These adaptations are amplified in individuals with multiple gestations^1^ and are of particular importance in patients with pre-existing cardiac disease who may not tolerate volume overload.

### Cardiovascular risk stratification

Several tools are available for risk stratification among individuals with pre-existing cardiovascular disease, including the CARPREG (Cardiac Disease in Pregnancy Study) II, ZAHARA (Zwangerschap bij Aangeboren HARtAfwijking Pregnancy in Women with Congenital Heart disease), and the modified World Health Organization classification. The CARPREG II study demonstrated that individuals with pre-existing cardiovascular disease were at risk for pulmonary edema as early as 13 weeks of gestation extending up to 24 weeks postpartum. Predictors of adverse outcomes included a history of prior cardiac events, baseline New York Heart Association (NYHA) functional class III-IV or cyanosis, the presences of a mechanical valve, ventricular dysfunction, high-risk left-sided valve disease or left ventricular outflow tract obstruction, pulmonary hypertension, coronary artery disease, high-risk aortopathy, lack of prior cardiac intervention, and late pregnancy assessment.^3^ As such, pregnancies among these individuals are particularly high risk and merit close multidisciplinary care and monitoring.

## B-type natriuretic peptides and N-terminal pro-B-type natriuretic peptide

The natriuretic peptides include the biologically active B-type natriuretic peptide (BNP) and the inert N-terminal pro-B-type natriuretic peptide (NT-proBNP); both arise from a common precursor peptide proBNP, liberated when the parent peptide is cleaved by corin and/or furin. Natriuretic peptides can be released in response to numerous stimuli but predominantly reflect cardiomyocyte stretch, which may occur due to structural heart disease. Because of their sensitivity for myocardial dysfunction, BNP and NT-proBNP have become gold standards in the biomarker diagnosis of heart failure.^4^ Since pregnancy represents a physiologic state characterized by a marked increase in circulating volume reflected by increases in cardiac chamber size, there have been several studies investigating the behavior of natriuretic peptides in pregnancy and early puerperium. A summary of BNP/NT-proBNP characteristics in normal pregnancy, preeclampsia, cardiovascular disease, and obesity is outlined in Table 1.

**Table 1:** B-Type Natriuretic Peptide Use During Pregnancy

Normal Pregnancy	Preeclampsia	PPCM	Pre-Existing Cardiomyopathy	Congenital Heart Disease	Valve Disease
General principles					
Stable through trimesters	↑ concentration compared to normotensive ↑ concentration with early-onset PE and greater disease severity Second trimester NT-proBNP value not predictive of future preeclampsia ↑ NT-proBNP concentration in the first trimester is associated with lower risk of HDP and future hypertension	↓ BNP and NT-proBNP at index diagnosis predictive of LV recovery over time as compared with ↑ index values ↑ concentration seen in heart failure associated with pregnancy without systolic dysfunction (not meeting criteria for PPCM), but to a lesser degree than in patients with reduced LVEF	↑ concentration among pregnant individuals with congenital heart disease vs normal pregnancy Retains negative predictive value	NT-proBNP concentration >128 pg/mL at 20 wk predictive of future events in CHD	No specific BNP data in pregnant patients with valve disease
Clinical recommendations					
BNP/NT-proBNP can be used to exclude heart failure as it has a high negative predictive value	Obtain measurements of BNP/NT-proBNP in patients with signs or symptoms of heart failure	Obtain measurements of BNP/NT-proBNP at the time of presentation for diagnosis and prognosis	Consider baseline and serial measurements of BNP/NT-proBNP in pregnant patients with cardiomyopathy who are at risk for heart failure (eg, systolic dysfunction)	Consider baseline and serial measurements of BNP/NT-proBNP in pregnant patients with congenital heart disease who are at risk for heart failure (eg, complex congenital lesions, systolic dysfunction)	Consider baseline and serial measurements of BNP/NT-proBNP in pregnant patients with valve lesions who are at risk for heart failure (eg, severe stenotic or regurgitant valve lesions, systolic dysfunction)

BNP = B-type natriuretic peptide; CHD = congenital heart disease; CVD = cardiovascular disease; HDP = hypertensive disorders of pregnancy; LVEF = left ventricular ejection fraction; NT-proBNP = N-terminal pro-B-type natriuretic peptide; PE = preeclampsia; PPCM = peripartum cardiomyopathy.

### Healthy pregnancy

Despite significant hemodynamic changes with pregnancy and normative cardiac chamber dilatation, BNP and NT-proBNP concentrations remain stable throughout the trimesters.^5^, ^6^, ^7^, ^8^, ^9^ In a series of 116 patients with serial BNP and NT-proBNP measured during pregnancy and the immediate postpartum period, natriuretic peptide levels increased within 48 hours postpartum, with a mean BNP of 85 ± 71 pg/mL and a mean NT-proBNP of 158 ± 167 pg/mL. Left ventricular and left atrial volumes similarly increased, but there were no differences in measures of diastolic function 48 hours postpartum as compared with the nonpregnant state and no reported adverse outcomes. Values returned to their presumed nonpregnant values (as assessed 6-12 months postpartum) by 6 to 12 weeks postpartum.^9^ In a study of 260 healthy pregnant individuals, Dockree et al reported a 95% upper reference limit for BNP of 50 pg/mL with no significant trimester-related differences. BNP degradation is influenced by neprilysin, which is released by the placenta into the maternal circulation. The authors proposed that because of excess placenta-derived circulating neprilysin, the sensitivity of BNP might be reduced and NT-proBNP (which is not a target of neprilysin) may be a more robust marker during pregnancy. More data regarding this interesting hypothesis are needed. The upper reference limit for NT-proBNP in the same study was 200 pg/mL in the first and second trimester and 150 pg/mL in the third, suggesting an effect of hemodilution with the increased volume of later pregnancy.^7^

### Use of natriuretic peptides for the assessment of heart failure in pregnancy

Both BNP and NT-proBNP retain their negative predictive value to exclude heart failure in pregnancy and peripartum period such that low concentrations help to exclude the diagnosis.^10^ However, the positive predictive value of natriuretic peptides for heart failure is generally lower. In one study of healthy pregnant individuals, mean BNP levels during late pregnancy were higher than nonpregnant values (18 pg/mL, 95% CI: 0-45 pg/mL vs 12 pg/mL, 95% CI: 0-28 pg/mL, *P* < 0.001) with a further transient increase to 43 pg/mL (95% CI: 0-113 pg/mL) early postpartum. At 1 month postpartum, levels remained higher than those of nonpregnant controls (16 pg/mL vs 12 pg/mL, *P* = 0.001). Of the 773 asymptomatic pregnant individuals studied, 6.1% had BNP levels exceeding 100 pg/mL (the conventional upper reference limit for heart failure) and 1% had BNP levels ≥200 pg/mL, all during the early postpartum period. Among individuals with elevated BNP values, one demonstrated echocardiographic evidence of diastolic dysfunction, but all others remained without decrement in systolic function or clinical heart failure. Concentrations of BNP were negatively correlated with body mass index (BMI) and hemoglobin and positively correlated with creatinine. Thus, while low BNP concentrations during pregnancy and puerperium can provide reassurance with respect to the absence of current clinical cardiovascular decompensation, significant elevations do not necessarily signify clinically evident ventricular dysfunction.^8^

### Preeclampsia

Several small studies have reported higher natriuretic peptide concentrations among pregnancies complicated by preeclampsia than among normotensive pregnancies^11^, ^12^, ^13^, ^14^, ^15^, ^16^ as shown in Tables 2 and 3. Individuals with early-onset preeclampsia and those with severe features exhibit higher values than those with milder disease and normotensive pregnancies.^12^^,^^15^ Studies further suggest a correlation between both NT-proBNP and BNP concentrations and echocardiographic assessments of left ventricular wall thickness and mass, and it has been hypothesized that elevated natriuretic peptide concentrations reflect subclinical left ventricular dysfunction among preeclamptic individuals.^11^^,^^14^^,^^16^ BNP levels at the time of hospital admission have also been found to be higher among preeclamptic individuals who develop pulmonary edema than among those who do not (55.4 pg/mL vs 42.0 pg/mL, *P* = 0.008).^12^

**Table 2:** BNP in Normal vs Preeclamptic Pregnancies

First Author (n)	Normal (pg/mL)	Late vs Early Preeclampsia (pg/mL)	
Borges et al^11^ 2018 (85)	43^a^	147^a^	214^a^
Resnik et al^15^ 2005 (118)	18^a^	21^a^	101^a^
Hameed et al^5^ 2009 (54)	19 (10-143)		
Burlingame et al^9^ 2017 (116)	25 (18-35)		
Dockree et al^7^ 2021 (260)	12 (0-52)		
Mayama et al^8^ 2017 (773)	18 (0-45)		
Tanous et al^10^ 2010 (78)	35 (21-43)		

Values are median (IQR)

a Interquartile range unavailable.

**Table 3:** NT-proBNP in Normal vs Preeclamptic Pregnancies

First Author (n)	Normal (pg/mL)	Any Preeclampsia (pg/mL)
Alvarez et al^13^ 2016 (n = 340)	49 (27-93)	101 (56-186)
Giannubulo et al^14^ 2017 (n = 148)	43 ± 11	121 ± 26
Rafik et al^16^ 2009 (65)	46 ± 6	477 ± 152
Burlingame et al^9^ 2017 (116)	36 (22-56)	
Dockree et al^7^ 2021 (260)	41 (13-155)	

Values are median (IQR) or mean ± SD.

### Postpartum preeclampsia

While most cases of preeclampsia are diagnosed during pregnancy, it is increasingly recognized that preeclampsia can develop de novo in the postpartum period (between 48 hours after delivery and up to 6 weeks postpartum). Data are more limited with respect to this recently appreciated entity; from a single institution (n = 22), median BNP measured at incident presentation was 424 pg/mL (interquartile range [IQR]: 190-645).^17^^,^^18^

### Natriuretic peptides as a predictor of preeclampsia

Whether natriuretic peptide levels in early pregnancy (prior to clinical disease manifestation) assist in prediction of future clinical trajectory has also been studied with mixed results. In a small study of individuals in their second trimester who subsequently developed preeclampsia (n = 16) compared with those whose pregnancies remained uncomplicated (n = 43), no difference emerged with respect to NT-proBNP concentration prior to disease manifestation (median: 52 vs 53 pg/mL).^19^ Data from the Nulliparous Pregnancy Outcomes Study (4,103 individuals, followed up for 2-7 years) showed that higher first trimester NT-proBNP concentrations were associated with a lower risk of hypertensive disorder of pregnancy and lower risk for development of hypertension 2 to 7 years postpartum. Potential mechanisms for these novel results suggest that elevated natriuretic peptides early in healthy pregnancy may represent physiologically appropriate first trimester volume expansion, which occurs to enable adequate uteroplacental blood flow, and reduced vascular resistance. The opposite would be proposed in those with lower values: vascular stiffness and less robust volume expansion.^20^

Other natriuretic peptides may be abnormal in hypertensive disorders of pregnancy. Lin et al reported that concentrations of N-terminal pro-atrial natriuretic peptide (NT-proANP) were significantly increased in hypertensive disorders of pregnancy. This inert N-terminal fragment of ANP is easier to measure (as ANP is rapidly degraded by neprilysin, which is in abundance in the circulation in the pregnant state). Notably, higher levels of corin (an enzyme involved in processing of both A- and B-type natriuretic peptides) were also elevated in those with hypertension and adverse pregnancy outcomes.^21^ These results support a role of natriuretic peptide release in hypertensive disorders of pregnancy, presumably identifying myocardial stress in this setting and predicting worse prognosis. In summary, while clinical judgment is necessary when interpreting any laboratory test, in the appropriate setting, the aggregated data would indicate elevated natriuretic peptide values may assist in the clinical evaluation of possible preeclampsia, identifying myocardial stress in this context and predicting risk for adverse pregnancy outcome.

### Peripartum cardiomyopathy

Natriuretic peptides elevate during incident heart failure from acute peripartum cardiomyopathy (PPCM) as with acute heart failure in the context of other dilated cardiomyopathies. PPCM patients who present with lower BNP (727 ± 634 pg/mL vs 26,887 ± 1,600 pg/mL, *P* < 0.001) concentrations at incident presentation are more likely to recover left ventricular function at 6 months after diagnosis of PPCM than those with higher levels, highlighting the potential prognostic value of natriuretic peptides.^22^^,^^23^ In a multicenter cohort study of individuals with PPCM, a median NT-proBNP concentration at diagnosis of <2,585 pg/mL was associated with a higher event-free survival than in individuals with higher values (*P* = 0.018).^24^ Interestingly, while PPCM is a disease of late pregnancy and early postpartum, a small study of first trimester biomarkers among women without pre-existing cardiovascular disease (CVD) found higher NT-proBNP levels (115.5 pg/mL [82.9-176.9]) among women who later developed PPCM (n = 4) than among those with healthy pregnancies both after matching for baseline demographic variables (n = 17, NT-proBNP 56.1 pg/mL [38.7-40.1]) and after matching additionally for blood pressure and pregnancy weight gain (n = 16, NT-proBNP 37.6 pg/mL [23.3-53.8]).^25^ However, further study is needed, potentially utilizing later gestational time points, to determine whether NT-proBNP may be able to identify women at elevated risk for future development of PPCM.

### Peripartum heart failure in the absence of LV dysfunction

It has now been increasingly recognized that heart failure can develop during pregnancy and the postpartum period even in the absence of LV dysfunction among individuals without pre-existing CVD.^26^, ^27^, ^28^, ^29^ During such acute heart failure presentations, BNP levels have been found to be elevated above normal values but to a lesser degree than patients who develop classic PPCM with systolic dysfunction (363 ± 270 vs 801 ± 416 pg/mL, *P* < 0.001).

### Pre-existing heart disease

Patients with pre-existing heart disease exhibit higher BNP values at all stages of pregnancy than pregnant individuals with structurally normal hearts.^10^ Further, individuals with pre-existing heart disease exhibit a rise in BNP between first and third trimesters—with a significant proportion exceeding 100 pg/mL—in contrast to normative pregnancies in which BNP concentrations remain stable and low throughout the trimesters.^10^ Nonetheless, natriuretic peptides retain their negative predictive value among individuals with pre-existing heart disease with a 100% negative predictive value for adverse cardiovascular events among individuals with a BNP value ≤100 pg/mL during pregnancy and a 96.9% negative predictive value for NT-proBNP values <128 pg/mL at 20 weeks of gestation in 2 studies. In addition, a 20-week NT-proBNP >128 pg/mL had value along with subpulmonary ventricular dysfunction and presence of mechanical valve for predicting adverse cardiovascular events.^10^^,^^30^ In the Standardized Outcomes in Reproductive Cardiovascular Care (STORCC) initiative, BNP was measured within 18 months preconception (when possible), at each trimester, and at postdelivery follow-up among patients with pre-existing CVD, the majority of which (90%) was comprised of congenital heart disease (CHD). Values above the reference range (as established for the nonpregnant population) were associated with a greater risk of adverse cardiac outcomes (43% vs 9%, *P* = 0.02 in this study of 184 individuals).^31^ Elevated BNP concentrations have also been associated with small-for-gestational-age infants in individuals with CHD.^32^ Given that there is often an overlap between symptoms of normal pregnancy (eg, fatigue, mild exertional dyspnea, mild lower extremity edema) and early signs of heart failure in particular, routine measurement of natriuretic peptides among patients at risk for development of heart failure during pregnancy may assist in the early detection of cardiovascular decompensation.

### Obesity and plasma natriuretic peptides pregnancy

It is well known that overweight and obese individuals have lower BNP and NT-proBNP values than do those with normal BMI.^33^ Lowering of BNP and NT-proBNP in obesity may result in lower sensitivity if using standard cutoffs in those with higher BMI; in one study, the diagnosis of heart failure was missed in one of 5 patients with a BMI ≥35 kg/m^2^.^34^ To address this issue, lower reference limits may be utilized in obesity; for example, guidelines from the European Society of Cardiology suggest a 50% reduction in cut points for BNP with obesity.^35^

The pathophysiologic mechanisms relating obesity and low concentrations of natriuretic peptides have not been fully elucidated. While some enhanced clearance of BNP through excess neprilysin production from adipose tissue might explain the finding, NT-proBNP remains unaffected and, therefore, may be of value in obese population. To the extent BNP and NT-proBNP arise from a common precursor, it implies the overwhelming majority of lowering is related to suppression of the NPPB gene by factors related to obesity, including hormonal changes co-segregating with increased adipose tissue, such as mediators of insulin resistance and androgen elevations.^36^, ^37^, ^38^

The effect of obesity during pregnancy on natriuretic peptide levels has not been well studied. A recent analysis of 260 healthy pregnant individuals sampled each trimester found significantly lower NT-proBNP values in overweight individuals in the third trimester when stratified by BMI <25 kg/m^2^ or >25 m^2^, but BMI was not stratified further.^7^ The relationship of BMI for pregnant individuals who are obese or morbidly obese remains a knowledge gap for future analysis.

### Natriuretic peptides and suggestions for clinical practice (Central Illustration)

1. In healthy asymptomatic pregnant individuals, natriuretic peptides should not be routinely measured.

**Central Illustration:**
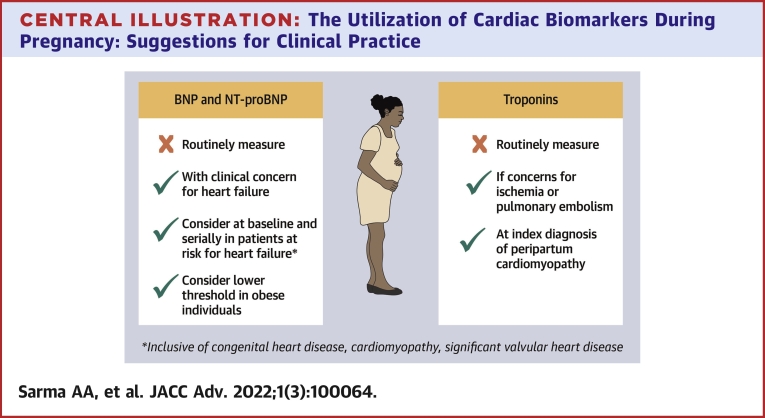
**The Utilization of Cardiac Biomarkers During Pregnancy: Suggestions for ClinicalPractice**
2. To prevent delayed diagnosis of heart failure, we suggest measuring natriuretic peptides in pregnant or postpartum individuals with new clinical symptoms or suggestive signs of heart failure
  a) The suggested upper reference limit for BNP is 50 pg/mL, with no significant trimester-related differences.
  b) The suggested upper reference limit for NT-proBNP is 200 pg/mL in the first and second trimester and 150 pg/mL in the third trimester and postpartum period.
3. In individuals with established cardiovascular disease who are at risk for heart failure during pregnancy (eg, CHD, pre-existing cardiomyopathy, significant valvular disease), measuring natriuretic peptides prior to conception (when feasible) or early in pregnancy, followed by serial natriuretic peptide measurement throughout each trimester and the early postpartum period, should be considered. Values should be measured in the event of symptoms suggestive of heart failure. Such approach may help guide clinical management and distinguish the symptoms of normal pregnancy from those of hemodynamic decompensation.
  a) Low concentrations (BNP [<100 pg/mL] or NT-proBNP [<128 pg/mL]) have a high negative predictive value; thus, concentrations below these thresholds make cardiovascular dysfunction unlikely, based on limited data.
  b) An elevated BNP or NT-proBNP should prompt further clinical evaluation, although elevations can be observed without clear clinical consequence. For such patients, an echocardiogram is a reasonable next step.
  c) Lower thresholds for abnormal natriuretic peptide levels should be considered with obesity, though there are very limited data to guide use during pregnancy. A normal value does not preclude the diagnosis of heart failure in the obese population and should be clinically correlated.

## Cardiac troponins

Biomarkers play an important role in detection of myocardial injury. Previously used biomarkers such as creatine kinase and its MB isoenzyme have poor specificity for myocardial injury in pregnancy and the postpartum period.^39^ On the other hand, troponin T (cTnT) and I (cTnI)—components of the cardiomyocyte contractile apparatus—have high specificity and have therefore supplanted creatine kinase in the diagnosis of acute myocardial injury. With a shift to assays providing high sensitivity at lower troponin concentrations, the fourth Universal Definition of Myocardial Infarction has articulated that myocardial injury describes a disorder of elevated troponin above the 99th percentile upper reference limit and is considered acute if associated with a rise and fall in values.^40^ Not only this has facilitated rapid evaluation of acute myocardial infarction (AMI) but also high-sensitivity cardiac troponin (hs-cTn) assays are increasingly utilized at a population level to screen for CVD and heart failure, where both hs-cTnI and hs-cTnT have been strongly associated with prevalent CVD/heart failure and also prognosticate their onset.^41^ Table 4 summarizes the characteristics of cardiac troponin in pregnancy.

**Table 4:** Cardiac Troponin Use During Pregnancy

Normal Pregnancy	Preeclampsia	Peripartum Cardiomyopathy	Pre-Existing Cardiomyopathy	Coronary Artery Disease	Valve Disease
General principles					
Stable through trimesters May be ↑ using high-sensitivity assays without clinical consequence in the early postpartum period A significant portion of patients will have values below the limit of detection using high-sensitivity assays	↑ concentration compared to normotensive pregnancies	Incident troponin values are inversely correlated with LV recovery	No specific data available in the pregnant population with cardiomyopathy	No specific data available in the pregnant population with pre-existing ischemic heart disease	No specific data available in the pregnant population with valve disease
Clinical recommendations					
↑ values should prompt investigation into potential ischemia	Obtain measurements of troponin in patients with signs or symptoms of ischemia	Obtain measurements of troponin at the time of presentation to aid with prognosis related to recovery of left ventricular systolic function	No data to support routine measurement of troponin in the absence of clinical concern for ischemia	No data to support routine measurement of troponin in the absence of clinical concern for ischemia Should be measured for acute chest pain or other clinical concern for ischemia both during pregnancy and the postpartum period (with the highest risk for P-SCAD occurring in the first postpartum week)	No data to support routine measurement of troponin in the absence of clinical concern for ischemia

↑ = increased/increase; P-SCAD = pregnancy-associated spontaneous coronary artery dissection.

### Normal pregnancy and preeclampsia

Current evidence suggests that conventional (non-high-sensitivity) cTn remains negative in normal pregnancies but may increase above the normal threshold in pregnancies complicated by preeclampsia although the data are inconsistent.^42^ A recent comprehensive review on cTn using non-high-sensitivity methods reported in uncomplicated pregnancy that the median cTnI was 30 ng/L (range: 10-300 ng/L), and in preeclampsia, it was 460 ng/L (range: 155-1,020 ng/L).^42^ Fleming et al^43^ reported a statistically significant increase in cTnI in individuals with gestational hypertension compared to normotensive individuals (median: 89 vs 30 ng/L, *P* < 0.001) and a subsequent increase in individuals with preeclampsia compared to gestational hypertension (155 vs 89 ng/L, *P* = 0.03).

Studies evaluating normative values of high-sensitivity assays in pregnancy and preeclampsia are limited. Prior studies to date have demonstrated that a significant portion of individuals with uncomplicated pregnancies have undetectable hs-cTnI concentrations.^25^^,^^44^ Among 880 pregnant individuals at various trimesters (predominately third), hs-cTnI was very low (median: 1.0 ng/L, IQR: 0.0-1.0) in 842 uncomplicated pregnancies but higher in those who developed preeclampsia (median: 12.0 ng/L, IQR: 3.0-97.5) or pregnancy-induced hypertension (11.0 ng/L, IQR: 6.0-22.3).^44^ Utilizing hs-cTnI, Morton et al found elevations above the upper limit of normal in 25% of preeclamptic individuals around the time of delivery, with normalization of values within 72 hours postpartum implying increased myocardial stress associated with preeclampsia; lending credence to this, concentrations of hs-cTnI further correlated with peak mean arterial pressure, though clinically evident cardiovascular dysfunction or myocardial ischemia was not evident. More data are needed in this area.^45^ Using a hs-cTnT assay in 150 healthy individuals, Smith et al^46^ reported a 4.3% incidence of concentrations in excess of the 99th percentile for a general, healthy population (14 ng/L) 8 to 24 hours postdelivery; considering sex-specific cutoffs for individuals are even lower (9 ng/L), the overall incidence of an abnormal hs-cTnT in this study was even higher. However, most of these elevated values were of no clinical significance and thus the etiology and interpretation of elevated hs-cTn remains uncertain in the context of uncomplicated labor. Further, the distribution of troponin elevations did not differ between individuals considered high vs low risk for cardiovascular complications.^46^

### Pre-existing cardiovascular disease

There are insufficient data with respect to troponin utilization among pregnant individuals with pre-existing cardiovascular pathology, including those with prior ischemic disease. In the absence of pregnancy-specific data, clinical inferences must be made from cardiovascular disease trends in the nonpregnant population. Regardless of the etiology, elevated troponin in the nonpregnant state is associated with increased mortality and adverse outcomes.^40^ Clinical correlation coupled with knowledge of the pathophysiology of cardiac biomarker release is essential to provide optimal perinatal care, while minimizing unnecessary, risky, or costly workup.

### AMI in pregnancy

While AMI occurs uncommonly during pregnancy, pregnancy substantially increases the risk of experiencing an AMI (3- to 4-fold), as well as associated mortality ∼11%.^47^ Similarly, pregnancy-associated spontaneous coronary artery dissection (P-SCAD) presents more aggressively than P-SCAD outside the context of pregnancy.^48^ As with the nonpregnant population, troponin is elevated in the context of AMI during pregnancy, though whether the degree of elevation differs as compared with the nonpregnant population is unknown. Given the significant morbidity and risk for mortality that AMI during pregnancy carries, an elevated troponin during pregnancy and the postpartum period (when most pregnancy-associated P-SCAD occurs) should prompt investigation into potential ischemia.

### PPCM

Limited data currently exist on the use of cardiac troponin in PPCM. One study of non-high-sensitivity cTnT in 106 patients with PPCM revealed that a troponin concentration >0.04 ng/mL at diagnosis was inversely predictive of left ventricular ejection fraction at 6 months (with a sensitivity of 54.9% and specificity of 90.9%).^49^ Given that hs-cTn is not confounded by obesity like BNP or NT-proBNP are, and the normal values for hs-cTn in healthy younger individuals are typically very low (implying even high-normal values might indicate pathology), there may be advantages to its measurement in pregnancy, particularly to identify and prognosticate acute diagnosis such as preeclampsia or PPCM. Additional studies are clearly needed.

### Cardiac troponins and suggestions for clinical practice (Central Illustration)

1. Troponin concentration should only be measured during pregnancy and the postpartum period in the context of clinical concern for myocardial ischemia or infarction.
2. Use of hs-cTN assays are the preferred test for evaluating pregnancy-related complications.
3. Elevated troponin concentrations should prompt further cardiovascular evaluation. It should be recognized that small increases may occur in the normal peripartum period and preeclampsia without known clinical significance with utilization of high-sensitivity assays. Despite this, abnormal concentrations of troponin are extremely rare in younger, healthy individuals and therefore warrant a full evaluation to rule out potential acute cardiovascular pathology.
4. Measurement of troponin at index PPCM diagnosis has prognostic implications for left ventricular recovery, based on limited studies.

## Special circumstances

### COVID-19 infection and pregnancy

Pregnant individuals are at an increased risk for severe complications from COVID-19 infection including intensive care unit admission, mechanical ventilation, and use of extracorporeal membrane oxygenation. Data from the living systematic review inclusive of 270,470 pregnant and postpartum mothers with confirmed or suspected COVID-19 found elevations in laboratory studies similar to the nonpregnant population, including 33% with lymphopenia, 28% with elevated white cell counts, 8% with thrombocytopenia, 32% with increased prolactin levels, and 51% with elevated C-reactive protein (CRP) levels. However, data regarding cardiac biomarkers in pregnancy are more limited.^50^ Mercedes et al studied 154 pregnant individuals with COVID infection at 32 ± 3.7 weeks of gestation and found that 15 (9.7%) had elevated NT-proBNP (median: 209 pg/mL, 184-246 pg/mL) and troponin I (median: 34.6 ng/mL, 14.4-55.5 ng/mL). All of these patients had abnormal findings on echocardiogram with mean left ventricular ejection fraction of 38%. In this cohort, all 15 individuals were critically ill and admitted to the intensive care unit, 13 were intubated and 2 died.^51^ This study, albeit small, suggests that similar to the nonpregnant population, biomarker elevation in the setting of COVID infection serves as a marker for adverse outcomes and mortality.^52^ A small study suggests that D-dimer is elevated in the context of COVID-19 during pregnancy as it is among nonpregnant individuals (n = 78), without difference between pregnant patients (n = 37) with mild vs severe disease (nonpregnant 1,610 μg/L [1,268-2,305], mild COVID pregnancy (1,654 μg/L [1,092-1,706]), and severe COVID pregnancy (1,729 μg/L [1,421-2,216], *P* = 0.49).^53^ Similar findings were reported in a larger (n = 227) study of pregnant and immediately postpartum women with COVID-19 infection in which D-dimer levels did not correlate with disease severity and need for supplemental oxygen.^54^

### Pulmonary embolism and D-dimer

Elevation of natriuretic peptides and troponin in nonpregnant patients with pulmonary embolism is associated with an increased risk of death and poor outcomes reflected by the presence of right ventricular strain and damage. Data among the pregnant population are lacking to determine whether this differs among the pregnant population. In the absence of data to suggest otherwise, elevated natriuretic peptides and troponin in the context of pulmonary embolism should raise concern for concomitant right ventricular strain.

D-dimer is often used in the nonpregnant population during the evaluation of suspected venous thromboembolism. During pregnancy, however, D-dimer increases (particularly in the third trimester) and declines rapidly postpartum (within 4-6 weeks).^55^, ^56^, ^57^ Normative trimester-specific values are <0.95 μg/mL for 1 to 13 weeks, <1.29 μg/mL for 14 to 26 weeks, and <1.7 μg/mL for 27 to 40 weeks.^58^ In the Diagnosis of Pulmonary Embolisms in Pregnancy (DIPEP) study of 310 pregnant patients with suspected pulmonary embolism, D-dimer levels were significantly higher among patients with venous thromboembolism than among those who did not, similar to the nonpregnant population.^59^ However, given elevations in D-dimer during normal pregnancy, standard cutoff values for the nonpregnant population cannot be utilized.^57^ In a prospective study of 510 pregnant individuals presenting with suspected pulmonary embolism, van der Pol et al examined a pregnancy-adapted YEARS algorithm in which 3 criteria were first considered: pulmonary embolism as the most likely diagnosis, clinical signs of deep vein thrombosis, and hemoptysis. Pulmonary embolism was safely excluded if none of these clinical criteria were met and the D-dimer was <1,000 ng/mL, or if one or more clinical criteria were met, the D-dimer was <500 ng/mL. Thus, low D-dimer levels may be able to assist in identification of women at low risk for pulmonary embolism (as with the nonpregnant population), but elevated levels merit further investigation if a clinical suspicion for venous thromboembolism is present.^60^

## Future perspective, evolving uses, and unknowns

The complex physiology of pregnancy creates a broad range of possible means by which to leverage biomarker measurement for monitoring of normative processes and prognosticate risk for pregnancy-associated complications such as gestational diabetes, fetal growth restriction, as well as certain hypertensive disorders of pregnancy including preeclampsia with or without superimposed chronic hypertension. Table 5 summarizes recently studied candidates for various applications.

**Table 5:** Novel Biomarkers for Various Pregnancy-Associated Diagnoses

Biomarker	Function	Application(s)?	Evidence
Adipokines			
Leptin	Regulates metabolism, weight regulation, and feeding	Prognosticate gestational diabetes, FGR, preeclampsia	Generally weak for all indications
Adiponectin	Mediates insulin sensitivity		
Visfatin	Mediates insulin sensitivity		
Resistin	Proinflammatory adipokine		
Irisin	Mediates exercise-related energy expenditures		
Omentin	Mediates insulin sensitivity		
Chemerin	Regulates glucose and lipid metabolism		
Apelin-1	Regulates vasomotor tone, insulin resistance		
PAPP-A	Placental function and fetal growth	Prediction of FGR	Modest evidence
First or second trimester free β-human chorionic gonadotropin	Hormone of pregnancy	Prediction of FGR	Weak, insensitive
α-Fetoprotein	Produced by fetal liver cells	Prediction of FGR	Weak, insensitive
Unconjugated estriol	Main estrogen of pregnancy	Prediction of FGR	Weak, insensitive
Inhibin A	Inhibits the synthesis and release of the follicle-stimulating hormone in the pituitary gland and reduces the hypothalamic LH-releasing hormone content	Prediction of FGR	Weak, insensitive
Angiogenesis-related markers			
sFlt-1	Antiangiogenic	Prediction/diagnosis of hypertensive disorders of pregnancy and FGR	Encouraging data for sFlt-1:PlGF ratio
PlGF	Proangiogenic		
Endoglin	Receptor for transforming growth factor β		
Metabolites	By-products of metabolism	Prediction of FGR	Insufficient data
RNA biomarkers (eg, micro-RNA)	Genomic regulation	Prediction of FGR, diagnosis of hypertensive disorders of pregnancy	Insufficient data
Proteomics	Patterns of biomarkers, including insulin-like growth factor acid–labile subunit and soluble endoglin, PlGF, serine peptidase inhibitor Kunitz type 1, melanoma cell adhesion molecule, selenoprotein P	Hypertensive disorders of pregnancy	Insufficient data

FGR = fetal growth restriction; PAPP-A = pregnancy-associated placental protein-A; PlGF = placental growth factor; sFlt-1 = soluble fms-like tyrosine kinase 1.

### Gestational diabetes mellitus

With rising incidence and prevalence of gestational diabetes mellitus, availability of a reliable biomarker approach to prognosticate onset and course of dysglycemia would be of interest. Although early research indicated that adipokines might be useful for prognosticating incident gestational diabetes (particularly measurement of chemerin), the data remain conflicting, and it is unlikely measurement of adipokines will gain traction for this indication.^61^

### Fetal growth restriction

Fetal growth restriction is associated with an increased risk for pregnancy loss and postnatal complications; accurate methods for its prediction are important yet remain limited. Adipokines have been measured for this purpose without significant evidence for utility.^62^^,^^63^ Other biomarkers have been explored for predicting fetal growth restriction; however, challenges remain with respect to timing (eg, which trimester to measure) and optimal cut points to determine an abnormal result. Among the markers examined are included pregnancy-associated placental protein-A, free β-human chorionic gonadotropin, α fetoprotein, unconjugated estriol, and inhibin A.^63^ Each of these is poorly sensitive but highly specific for fetal growth restriction; of the candidates, pregnancy-associated placental protein-A appears most promising but remains relatively insensitive. Emerging data suggest measurement use of omics approaches to quantify metabolites or members of the transcriptome such as micro-RNA might enhance ability to predict fetal growth restriction; however, these data remain preliminary.^64^^,^^65^

### Angiogenesis-related factors

Abnormalities of placental function are associated with risk for hypertensive disorders of pregnancy.^66^ Not only angiogenesis-related factors, including soluble fms-like tyrosine kinase 1 and placental growth factor, are participants in placental dysfunction but also evidence suggests their measurement is significantly associated with the ability to predict risk for preeclampsia.^67^ For example, when measured during the first trimester, the soluble fms-like tyrosine kinase 1-to-placental growth factor ratio may provide important information regarding subsequent development of preeclampsia, and later in pregnancy, the panel of these 2 biomarkers may be useful to diagnose preeclampsia in symptomatic mothers (including recognition of preeclampsia in individuals with antecedent gestational hypertension).^67^ Abnormal balance in these biomarkers of the “angiogenic placental syndrome” may also provide information regarding fetal growth restriction^64^ as well as risk for cardiomyopathy.

### Inflammatory biomarkers

Measurement of biomarkers associated with inflammation may have a role in several circumstances in pregnancy. For example, clinically, elevated concentrations of CRP in early pregnancy may be associated with fetal growth patterns and risk for neonatal complications.^68^ Additional roles for CRP measurement might include its application for assessment of risk for adverse outcomes of placental or respiratory infection from novel coronavirus.^69^ Lastly, emerging data focused on measurement of small, noncoding fragments of ribonucleic acid known as micro-RNA has revealed further connection between inflammatory processes and adverse pregnancy outcomes.^69^ More data are needed regarding measurement of inflammatory markers or mediators in pregnancy.

### Proteomics

Though the topic exceeds the scope of the discussion, use of targeted or untargeted proteomics to simultaneously measure hundreds to thousands of proteins in the pregnant state may be useful for discovery of previously unsuspected biological pathways of disease and potentially identify newer biomarkers for clinical measurement.^70^^,^^71^ As a proof of concept, when studying the proteome of women early in pregnancy, Myers et al^72^ identified biomarker combinations centered around insulin-like growth factor acid–labile subunit able to predict later risk for preeclampsia. More data are needed regarding the potential role of various omics (genomics, transcriptomics, proteomics, metabolomics) in better understanding normal and complicated pregnancies.

## Conclusions

Cardiac biomarkers have well established roles in the diagnosis, management, and risk stratification for patients with cardiac dysfunction, outside of pregnancy. Pregnancy imposes a remarkable cardiovascular hemodynamic burden, and even normal pregnancy can cause symptoms that would otherwise be consistent with acute cardiovascular pathology. As a result, cardiac biomarkers are a promising tool to help guide and expedite care in the pregnant population. Larger-scale studies are required to better characterize the clinical utilization of cardiac biomarkers, particularly the natriuretic peptides and troponin during pregnancy and the peripartum period both in the context of complicated and uncomplicated pregnancy. Further, several novel markers have been investigated but require further research with respect to their clinical application for pregnant individuals.

## Funding support and author disclosures

Dr Sarma has received the CRICO patient safety award and the MGH Department of Medicine Innovation grant. Dr Januzzi has received the Hutter Family Professorship; is a trustee of the American College of Cardiology; is a board member of Imbria Pharmaceuticals; has received grant support from 10.13039/100014386Abbott Diagnostics, Applied Therapeutics, Innolife, and 10.13039/100004336Novartis; has received consulting income from Abbott Diagnostics, Boehringer Ingelheim, Jana Care, Janssen, Novartis, Prevencio, and Roche Diagnostics; and participates in clinical end point committees/data safety monitoring boards for AbbVie, Siemens, Takeda, and Vifor. Dr Quesada has received support from the 10.13039/100000002NIH (K23-HL151867). Dr Briller is an unpaid consultant for the Illinois Maternal Mortality Committee; and is on the Steering committee for the REBIRTH trial of bromocriptine in Peripartum Cardiomyopathy (NCT05180773). All other authors have reported that they have no relationships relevant to the contents of this paper to disclose.
